# The Consortium for Clarity in ADRD Research Through Imaging (CLARiTI)

**DOI:** 10.1002/alz.14383

**Published:** 2024-11-26

**Authors:** Elizabeth C. Mormino, Sarah A. Biber, Annalise Rahman‐Filipiak, Konstantinos Arfanakis, Lindsay Clark, Jeffrey L. Dage, John A. Detre, Bradford C. Dickerson, Michael C. Donohue, Steven Kecskemeti, Timothy J. Hohman, William J. Jagust, Dirk C. Keene, Walter Kukull, Swati R. Levendovszky, Howie Rosen, Paul M. Thompson, Victor L. Villemagne, David A. Wolk, Ozioma C. Okonkwo, Gil D. Rabinvovici, Monica Rivera‐Mindt, Tatiana Foroud, Sterling C. Johnson

**Affiliations:** ^1^ Department of Neurology and Neurological Sciences Stanford University School of Medicine Palo Alto California USA; ^2^ Wu Tsai Neurosciences Institute Stanford University School of Medicine, Cogen Facility Stanford California USA; ^3^ National Alzheimer's Coordinating Center University of Washington Seattle Washington USA; ^4^ Department of Psychiatry University of Michigan Ann Arbor Michigan USA; ^5^ Department of Biomedical Engineering Illinois Tech Chicago Illinois USA; ^6^ Department of Medicine University of Wisconsin School of Medicine and Public Health, Health Sciences Learning Center Madison Wisconsin USA; ^7^ Department of Neurology Indiana University School of Medicine Indianapolis Indiana USA; ^8^ Stark Neurosciences Research Institute Indiana University School of Medicine Indianapolis Indiana USA; ^9^ Department of Neurology Perelman School of Medicine University of Pennsylvania Philadelphia Pennsylvania USA; ^10^ Department of Neurology Massachusetts General Hospital, Harvard Medical School Boston Massachusetts USA; ^11^ Department of Neurology Keck School of Medicine, University of Southern California Los Angeles California USA; ^12^ Alzheimer's Therapeutic Research Institute (ATRI), University of Southern California San Diego California USA; ^13^ Department of Neurology Vanderbilt University Medical Center Nashville Tennessee USA; ^14^ Department of Epidemiology School of Public Health University of California Berkeley California USA; ^15^ Helen Wills Neuroscience Institute University of California Berkeley California USA; ^16^ Department of Laboratory Medicine and Pathology University of Washington Seattle Washington USA; ^17^ Department of Epidemiology University of Washington Seattle Washington USA; ^18^ Department of Radiology University of Washington Seattle Washington USA; ^19^ Department of Neurology University of California San Francisco California USA; ^20^ Department of Ophthalmology, Psychiatry and the Behavioral Sciences, Radiology Psychiatry, and Engineering, Keck School of Medicine, University of Southern California Los Angeles California USA; ^21^ Department of Psychiatry University of Pittsburgh Pittsburgh Pennsylvania USA; ^22^ Department of Psychology Fordham University Bronx New York USA; ^23^ Department of Neurology Icahn School of Medicine at Mount Sinai New York New York USA; ^24^ Department of Medical and Molecular Genetics Indiana University School of Medicine Indianapolis Indiana USA

**Keywords:** Alzheimer's disease, biomarkers, magnetic resonance imaging, neuroimaging, pathology, plasma markers, positron emission tomography

## Abstract

The presence of multiple pathologies is the largest predictor of dementia. A major gap in the field is the in vivo detection of mixed pathologies and their antecedents. The Alzheimer's Disease Research Centers (ADRCs) are uniquely positioned to address this gap. The ADRCs longitudinally follow ≈ 17,000 participants, ranging from cognitively unimpaired to dementia, arising from Alzheimer's disease (AD) and related dementias (ADRD; e.g., AD, Lewy body disorders, vascular). Motivated by the Alzheimer's Disease Neuroimaging Initiative's (ADNI) impact, the ADRC Consortium for Clarity in ADRD Research Through Imaging (CLARiTI) was formed. Leveraging existing ADRC infrastructure, CLARiTI will integrate standardized imaging and plasma collection to characterize mixed pathologies and use community‐engaged research methods to ensure that ≥ 25% of the sample is from underrepresented populations (e.g., ethnoculturally minoritized, low education). The resulting ADRD profiles, within a more diverse sample, will provide key resources for ADRCs and an unprecedented, more generalizable publicly available imaging‐plasma dataset.

**Highlights:**

- In vivo detection of mixed pathologies is critical for Alzheimer's disease and related dementias research.
- The Alzheimer's Disease Research Centers (ADRCs) are uniquely positioned to address gaps related to mixed pathologies.
- The ADRC Consortium for Clarity in ADRD Research Through Imaging (CLARiTI) will enhance this national program by adding standardized imaging and plasma collection to existing ADRC infrastructure.
- This effort will provide key resources for ADRCs and an unprecedented publicly available imaging–plasma–neuropath dataset.

## 1 BACKGROUND

It is increasingly recognized that the clinical manifestation of presumed Alzheimer's disease (AD) is typically a mix of etiologies and that the presence of multiple etiologies is the norm among individuals with underlying AD pathology.^1^, ^2^, ^3^, ^4^ Even so, we lack the ability to identify mixed pathologies and antecedent states in vivo that present clinically as AD. Even among 70‐year‐old individuals with AD clinical syndrome, concomitant Lewy body (LB) proteinopathy, or/and limbic‐predominant age‐related TDP‐43 encephalopathy (LATE) are present 58% and 25%^1^, ^2^ of the time, respectively. Interestingly, the degree of co‐occurrence between AD pathology and LB pathology remains consistent across 60‐ to 90+‐year‐olds, whereas the co‐occurrence with LATE increases systematically with older age.^2^ Among Alzheimer's Disease Research Center (ADRC) subjects with AD neuropathology, concomitant cerebrovascular disease (CVD or V) is common (61%) and accelerates cognitive decline.^5^ There is an urgent need to address this gap, as greater co‐pathology is strongly associated with the degree of cognitive impairment^1^, ^6^, ^7^ and potentially with the pace of cognitive decline. Failure to account for co‐pathologies will result in inaccurate diagnoses and estimates of risk.^2^ This need is further accentuated with the emergence of anti‐amyloid disease‐modifying therapies for which the modulating effects of co‐pathologies on outcomes is unknown.

**FIGURE 1:**
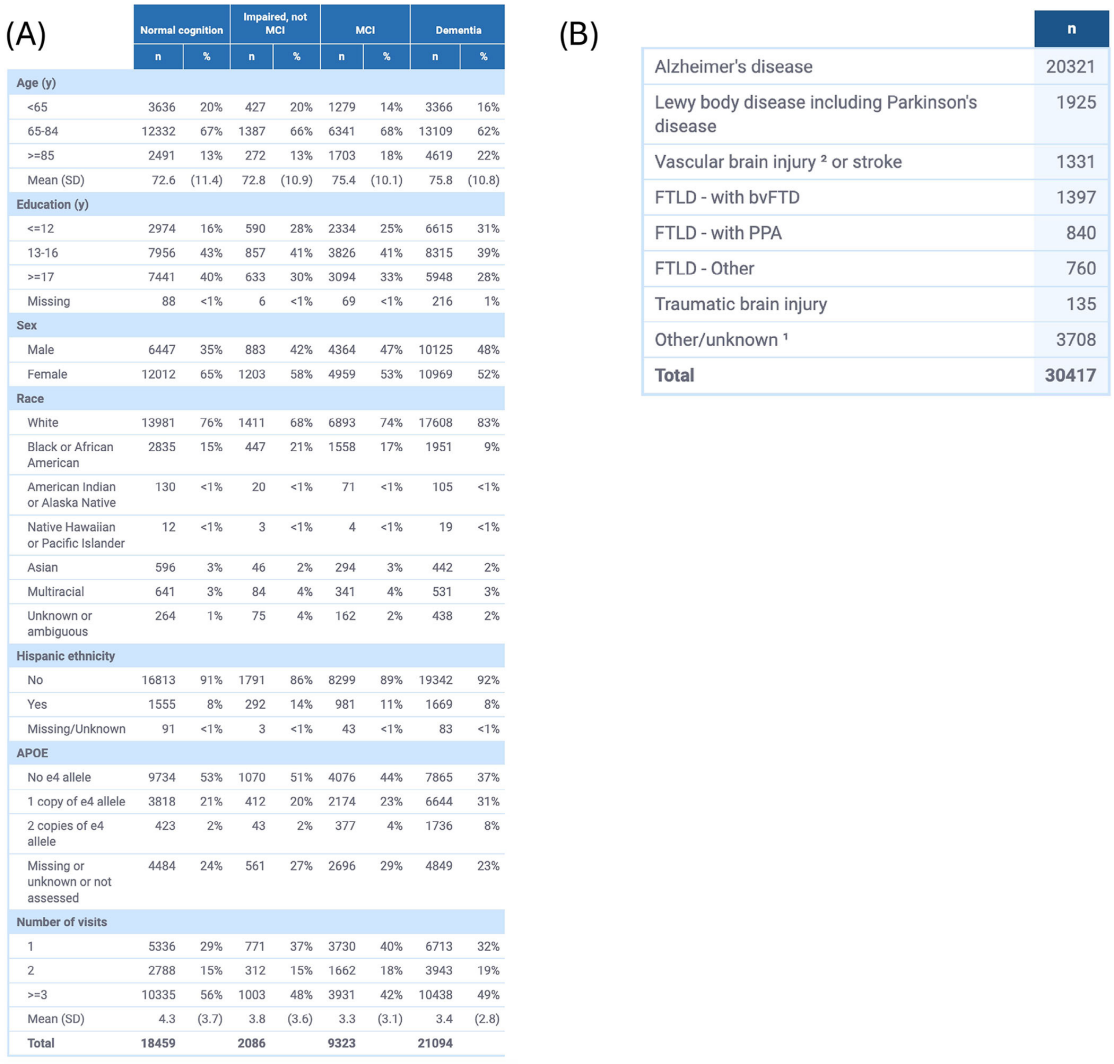
Participant characteristics for Clinical Core participants combined across all centers. Demographics are shown in (A) and primary suspected etiology underlying cognitive impairment is shown in (B). *APOE*, apolipoprotein E; bvFTD, behavioral variant frontotemporal dementia; FTLD, frontotemporal lobar degeneration; MCI, mild cognitive impairment; PPA, primary progressive aphasia; SD, standard deviation.

**FIGURE 2:**
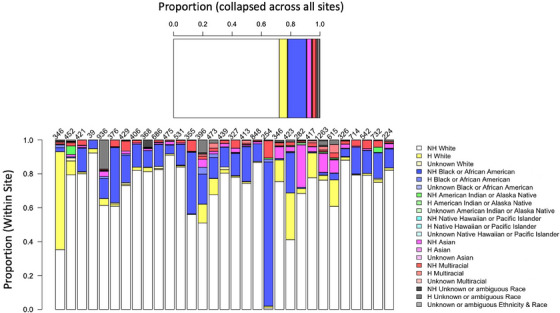
Self‐reported race and ethnicity across all active Clinical Core participants combined across centers (A) as well as within each center (B). Each column in (B) reflects a single site. The total number of active participants per center is shown at the top of each column. H, Hispanic; NH, non‐Hispanic.

The ADRC program is uniquely positioned to address gaps related to in vivo assessment of mixed pathologies given the inclusion of the full spectrum of AD and related dementias (ADRD) in ADRC cohorts. The ADRC program has been supported by the National Institute on Aging (NIA) since 1984 and is currently a collection of ≈ 35 individual sites across the United States. These sites are supported by numerous infrastructure initiatives that collectively support site collaboration, multimodal data storage and analysis, as well as data dissemination (Table 1). The National Alzheimer's Coordinating Center (NACC) functions as the centralized data repository for the ADRC program, and additionally provides infrastructure for collaboration and communication across sites.^8^, ^9^, ^10^ The overarching goal of the ADRC program is to improve the diagnosis and care for individuals with ADRD by providing core resources to enhance local research at each institution as well as to contribute to national collaborative efforts. The focus of each individual ADRC varies depending on the institution: some sites focus on AD itself or risk factors for AD, while others focus on vascular contributions to dementia, the LB spectrum, TDP‐43, atypical dementias or neurobehavioral presentations, and so on. This range of expertise and focus ensures that, in aggregate, the entire spectrum of etiologies underlying ADRD are represented across the program. There is also valuable demographic variability in the characteristics of Clinical Core cohorts across sites, with many sites having expertise in the inclusion of individuals typically underrepresented in ADRD research programs based on race, ethnicity, rurality, and/or socioeconomic status (SES). These characteristics have resulted in the ADRCs collectively following the largest known ADRD cohort (Figure 1). The ADRCs longitudinally collect standardized rich multi‐domain neurocognitive clinical and phenotypic data (Uniform Data Set [UDS], in place since 2005)^8^, ^11^ that are made publicly available via NACC. To date, there is UDS data available for > 50,000 unique Clinical Core participants, 17,000 of whom are currently active participants. Each ADRC has a specialty focus within ADRD and a cohort that, on average, is ≈ 420 active participants who are followed annually or biannually with standard clinical/cognitive assessments using the UDS. Across all sites, 27% of active participants are from underrepresented populations (URPs) based on race and ethnicity (Figure 2). A unique feature of the ADRC program is the high rate of brain donor enrollment and autopsy success rates (each near 60%). There are currently > 7800 participants with pathology data and *ante mortem* UDS clinical/cognitive data.^10^ The National Centralized Repository for Alzheimer's Disease and Related Dementias (NCRAD) supports the ADRCs with a fluid biorepository, genome‐wide panels, and standard blood collection procedures.

**TABLE 1:** ADRC program components.

	Description
Alzheimer's Disease Research Centers (ADRCs)	Individual centers throughout the United States, funded via a project grant by the National Institute on Aging (NIA). Each ADRC enrolls a Clinical Core cohort relevant for ADRD. Inclusion is determined based on center expertise.
National Alzheimer's Coordinating Center (NACC)	NACC is the centralized data repository for the ADRC program, and additionally provides infrastructure for collaboration and communication across sites. NACC is directed by Drs. Walter Kukull and Sarah Biber at the University of Washington.
National Centralized Repository for Alzheimer's Disease and Related Dementias (NCRAD)	NCRAD supports the ADRCs with a fluid biorepository, genome‐wide panels, and standard blood collection procedures. NCRAD is directed by Dr. Tatiana Foroud at Indiana University.
Standardized, Centralized AD/ADRD Neuroimaging (SCAN)	SCAN implements multi‐site workflows for standardized acquisition and analysis of PET and MRI data across ADRC sites. The SCAN PET Core is led by Bill Jagust at University of California Berkeley and the SCAN MRI Core is led by Clifford Jack at Mayo.
The Laboratory of Neuroimaging (LONI)	LONI acts as the repository for PET and MRI image data collected with SCAN standardized protocols across the ADRC sites. LONI is directed by Dr. Arthur Toga at the University of Southern California.

Abbreviations: ADRD, Alzheimer's disease and related dementias; MRI, magnetic resonance imaging; PET, positron emission tomography.

**FIGURE 3:**
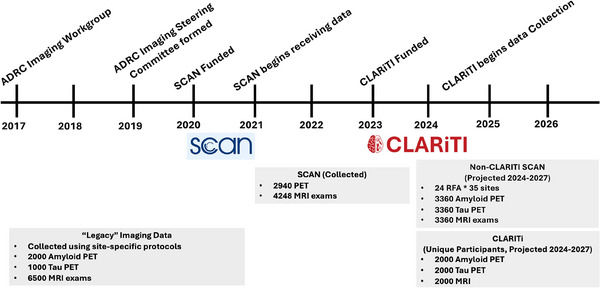
Timeline describing integration of coordinated imaging efforts within the ADRC program. Current and estimated projections are listed for each imaging category. ADRC, Alzheimer's Disease Research Center; CLARiTI, ADRC Consortium for Clarity in ADRD Research Through Imaging; MRI, magnetic resonance imaging; PET, positron emission tomography; RFA, request for application; SCAN, Standardized, Centralized AD/ADRD Neuroimaging.

The ADRC program has recently launched multiple efforts geared toward standardized in vivo biomarker data across sites (Figure 3). In particular, standardized amyloid–tau–neurodegeneration (ATN) imaging (amyloid and tau positron emission tomography [PET], alongside magnetic resonance imaging [MRI] to capture neurodegeneration) was not collected uniformly across ADRCs until 2021. Prior to this, ATN imaging on ADRC Clinical Core participants was largely performed in the context of investigator‐led research grants using local imaging protocols (“legacy” imaging data). These ATN imaging studies are a clear example of the success of the ADRC program in enhancing local investigator‐led science, resulting in key publications that significantly contributed to the development of current ATN research frameworks for the biological staging of AD (for example, Klunk et al.^12^) used Pittsburgh ADRC brain tissue samples for autoradiography studies to validate Pittsburgh compound B (PiB) PET imaging; Jack et al.^13^ recruited Mayo ADRC Clinical Core participants to produce one of the initial papers that characterized amyloid and neurodegeneration profiles). However, ad hoc imaging performed through investigator‐initiated studies results in data captured incompletely and with inconsistent protocols across Clinical Core participants, as the recruitment strategy and imaging schedule are determined by the specific research grant funding the studies. Critically, these efforts have resulted in only a small subset of Clinical Core participants characterized with ATN imaging (in a 2022 survey developed by the ADRC Imaging Core Steering Committee and completed across 37 ADRC sites, on average, only 20% of active Clinical Core participants had undergone amyloid PET). Another limitation of this approach to imaging is inconsistencies in acquisition protocols across sites, complicating efforts to harmonize data across centers. Finally, although data sharing is a priority for all National Institute of Health (NIH)‐funded efforts, the procedures for data sharing are site specific and dependent on criteria detailed in study‐specific consent forms. Although a subset of sites have voluntarily uploaded their imaging data to NACC for widespread distribution, these data likely reflect only a small portion of the actual imaging data that have been captured on Clinical Core participants, limiting data leveraging opportunities across centers.

In 2017, an ADRC workgroup comprised of neuroimaging experts and key stakeholders recommended that the ADRCs shift toward collecting and sharing standardized PET and MRI data across sites. In 2020, the NIA established a resource (U24 AG067418 to Jagust and Jack) called Standardized, Centralized AD/ADRD Neuroimaging (SCAN), which functions in collaboration with the ADRCs, the Laboratory of Neuroimaging (LONI, at the University of Southern California), and NACC. It receives quality checks, processes, harmonizes, and makes publicly available the raw and processed images contributed by the centers and the quantified output via the NACC Data Platform. The repository at LONI that hosts these standardized images became active in January 2021. SCAN developed a standard imaging protocol through a 2‐year consensus process with the centers, which mirrors ADNI MRI and PET protocols. Although SCAN provided an important infrastructure to support standardized image collection and analysis, this initiative did not include funding for image acquisition or specify a recruitment strategy for addressing specific scientific goals. The ADRC Consortium for Clarity in ADRD Research Through Imaging (CLARiTI) was formed to secure these necessary resources and provide a collaborative platform to improve in vivo detection of mixed pathologies.

**FIGURE 4:**
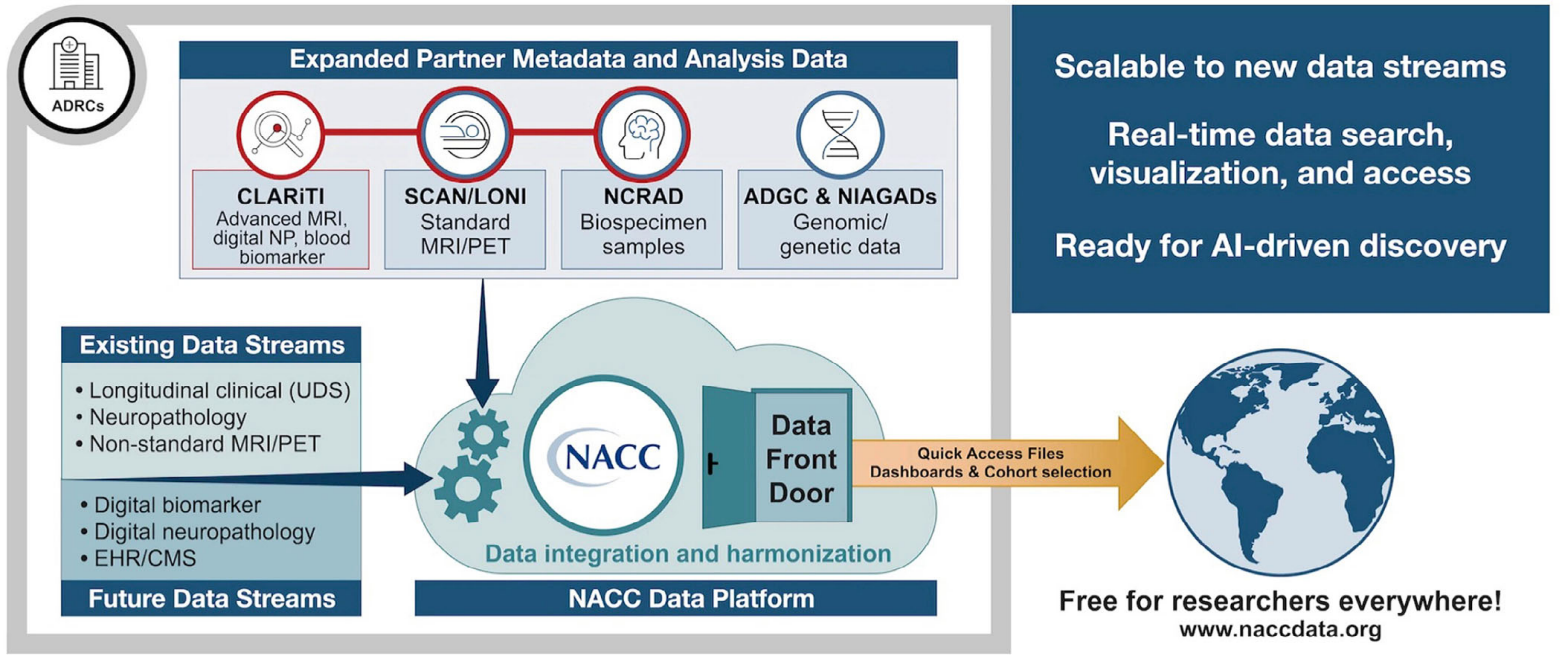
Summary of ADRC infrastructure and data streams relevant for wide‐scale data sharing. ADGC, Alzheimer's Disease Genetics Consortium; ADRC, Alzheimer's Disease Research Center; AI, artificial intelligence; CLARiTI, ADRC Consortium for Clarity in ADRD Research Through Imaging; CMS, Centers for Medicare and Medicaid Services; EHR, electronic health record; LONI, Laboratory of Neuroimaging; MRI, magnetic resonance imaging; NACC, National Alzheimer's Coordinating Center; NCRAD, National Centralized Repository for Alzheimer's Disease and Related Dementias; NIAGADS, National Institute on Aging Genetics of Alzheimer's Disease Data Storage Site; PET, positron emission tomography; RFA, request for application; SCAN, Standardized, Centralized AD/ADRD Neuroimaging; UDS, Uniform Data Set.

The initial idea to form an imaging consortium emerged in 2021 during conversations within the ADRC Imaging Core Steering Committee. We sought to increase the resources for neuroimaging in the ADRC program, while at the same time leveraging SCAN, the NACC Data Platform, and the entire ADRC ecosystem, maximizing the investments that NIA had already made (Figure 4). We argued that in vivo characterization of Clinical Core participants from this effort would benefit local institutional research as well as create an unprecedented public dataset for the scientific community to address questions related to mixed pathologies. Scientific aims include the characterization of disease burden for AD and ischemic small vessel disease, the two most common diseases in the aging brain. In the future, modeling techniques will be applied to amyloid, tau, and vascular ischemic lesion burden assessments to identify the presence of biomarker abnormalities, and, when abnormal, also provide estimated onset ages and durations.^14^, ^15^, ^16^, ^17^ We will also estimate the likelihood of additional co‐pathology, such as other LB disorders and LATE, based on the presence, proportion, and spatial patterns of imaging (e.g., mismatch^18^) and plasma indicators of neurodegeneration relative to AD burden. Overall, this project addresses the urgent need to assess the consequences and extent of mixed pathologies in ADRD research programs, provides a framework to validate imaging and plasma biomarkers with eventual neuropathology, and contributes to wide‐scale data sharing.

## 2 STUDY PROTOCOL

CLARiTI is a 5‐year project that was funded in 2023 (U01‐AG082350), with participant enrollment beginning in mid‐2024. CLARiTI is an observational longitudinal ATN imaging and blood collection study superimposed on the standard data collection protocols already in place at each ADRC. Enrollees into CLARiTI will complete amyloid PET, tau PET, MRI, and blood draw at two time points separated by a target of 2 years. To avoid redundant procedures, MRI and plasma collection may be waived if already collected through separate “compliant” protocols within 12 months of CLARiTI PET scanning (SCAN compliant MRI and the Alzheimer's Disease Center Fluid Biomarkers (ADCFB) protocol for blood). These imaging–plasma sets will, in the future, be intrinsically linked to cognitive and neurobehavioral data collection, genetics, and eventual neuropathology (Figure 3).

The primary goal of CLARiTI is to enhance the ADRC program by supporting ATN characterization of Clinical Core participants. Accordingly, all participants who enroll in CLARiTI will be Clinical Core participants at their local ADRC. The overall enrollment goal is to include 2000 participants age ≥ 55 who are either clinically unimpaired (≈ one third) or have a clinical impairment (mild cognitive impairment [MCI] or dementia, ≈ two thirds). As part of CLARiTI, these participants will undergo baseline imaging as well as repeat imaging 2 to 3 years after their initial visit. Longitudinal clinical and cognitive data will be collected as part of their site‐specific ADRC visits. Of those that are clinically unimpaired, one or more risk factors for dementia should be present (age > 75, member of an URP, low education, apolipoprotein E [*APOE*] ε4 positivity, family history of dementia, vascular risk factors, cognitive complaints, or neurobehavioral features such as mild behavioral impairment). For participants with clinical impairment, underlying AD should be suspected, even if in conjunction with another primary driver of impairment (for instance, a participant who is clinically suspected of having underlying LB pathology may also be suspected of having AD co‐pathology).

Enrollment procedures into CLARiTI across different ADRC Clinical Cores will differ across sites. For example, many sites actively recruit new participants into their centers, whereas some sites focus mostly on longitudinal follow‐ups of already enrolled participants. This variability in focus on enrollment versus retention is related to a number of factors, with one major factor being the maturity of the site (some sites have been active for decades whereas others are recently funded centers). As most of the current Clinical Core participants have never been characterized with ATN imaging, enrolling these pre‐existing participants into CLARiTI represents an ideal opportunity to enhance the characterization of those who have already been deeply phenotyped from a clinical perspective and may have various additional data already collected at their local ADRC (legacy imaging, plasma, cerebrospinal fluid [CSF], etc.). CLARiTI participants can be newly enrolled or may also have been a part of their local ADRC for many years. Each site will be responsible for deciding what inclusion strategy is optimal for their site's CLARiTI goals (see the Inclusion Core section below for recruitment resources that will be provided for sites if needed).

Participants who are co‐enrolled in the Alzheimer's Disease NeuroImaging Initiative (ADNI), Longitudinal Early‐Onset Alzheimer's Disease Study (LEADS), or other studies that already involve amyloid and tau PET scanning would not be appropriate for CLARiTI because these participants already have ATN characterization and those data are already publicly available. Enrollment into any of these studies is part of the exclusion criteria for the CLARiTI study. In 2023, new requirements were implemented by NIA that require ADRCs to collect 24 SCAN‐compliant ATN imaging sets on their Clinical Core participants per year as part of their center budget (applying to both new centers and as established centers are renewed). These 24 ATN imaging sets are separate from CLARiTI scans, though they will be easily combined for analysis because they will be following the same SCAN‐compliant acquisition protocols. Collectively, we anticipate an average of 24 ATN sets collected outside CLARiTI and another 24 ATN collected through CLARiTI per year. This amounts to ≈ 1600 to 1700 Clinical Core participants per year across efforts within the ADRC program.

A single institutional review board will be used across all sites for the CLARiTI study. All participants enrolled in CLARiTI will provide informed consent or assent if unable to provide consent (in which case a surrogate will provide consent). De‐identified data collected through CLARiTI will be shared broadly with the scientific community via NACC.

### 2.1 Standard data already collected on ADRC participants

CLARiTI focuses on the integration of standardized imaging and plasma data collection into the ADRC program. This project will be superimposed on the clinical data collection already in place at each ADRC. Clinical data are collected via the UDS, which includes deep clinical characterization across both cognitive and functional levels. Additional modules are collected depending on the site expertise (frontotemporal dementia module, LB module, etc.). The imaging and plasma datasets collected through CLARiTI will be easily integrated with these ongoing clinical assessments at the individual site level, as well as through the data access platform within NACC (Figure 4).

### 2.2 SCAN‐compliant imaging acquisitions

Each site will follow the SCAN site qualification process for both PET and MRI. Sites are currently collecting SCAN‐compliant data through investigator‐funded initiatives, with > 2900 PET scans and 4200 MRI sessions collected between 2021 and April 2024 (at the time of writing). The additional SCAN‐compliant data collected through CLARiTI will follow the same data flow and processing procedures already in place within the SCAN PET and MRI Cores (see Landau et al. in this special issue).^19^ This site qualification process is identical to that of the ADNI study and has already been completed by the majority of the ADRC sites. This qualification process is overseen by the SCAN PET and MRI Cores. Amyloid and tau PET imaging will be collected for each participant using the site's preferred ligand and following the compliant acquisition windows and reconstruction parameters specified by SCAN. Although multiple amyloid and tau PET ligands are used across sites and in some situations within the site, longitudinal scans should be completed using the same radiotracers as the baseline visit for a given participant.

All CLARiTI PET data will be processed alongside other SCAN PET data collected outside the CLARiTI grant (see Landau et al. in this special issue). In brief, this processing provides a quantitative tracer‐specific dichotomous variable for amyloid+ versus amyloid−, a status that is identical to the amyloid status variable generated for ADNI PET data.^20^, ^21^ Continuous standardized uptake value ratios (SUVRs) are also generated for regions from the Global Alzheimer's Association Interactive Network (GAAIN) atlas (amyloid PET only)^22^ as well as for the Desikan–Killiany FreeSurfer atlas (amyloid and tau PET).^20^ Additionally, global SUVRs from the GAAIN atlas will be converted to Centiloids (CLs) using standard procedures.^22^

3T MRI scanning will be performed on the same or different days within ≈ 6 months of amyloid PET. Individuals who are ineligible, contraindicated, or unwilling to complete the MRI, but otherwise meet the eligibility criteria for this study, may still enroll at the discretion of the site lead researcher. The core SCAN‐compliant MRI protocol includes the SCAN T1‐weighted 3D volume to assess gray matter integrity and the SCAN T2‐weighted fluid‐attenuated inversion recovery (FLAIR) to enable quantification of white matter hyperintensities (referred to as ‘‘Option 1″). Additional sequences include a multi‐echo gradient echo T2* scan to assess microbleeds from the susceptibility weighting and also derive a quantitative susceptibility mapping (QSM) composite to address hypotheses related to neurodegeneration. All SCAN‐compliant sequences are intentionally identical to the current ADNI4 MRI protocol. Especially relevant for CLARiTI—given the high degree of overlap between AD and LB disease—QSM from the multi‐echo 3D T2* GRE MRI has shown signal increases across clinical stages of LB disease in the substantia nigra (SN).^23^ Thus, QSM may be a promising tool for capturing neurodegeneration in the context of LB disease, which is often co‐morbid with AD.

### 2.3 Additional neurodegeneration‐focused MRI

In addition to SCAN‐compliant MRI data, CLARiTI will support the acquisition of optional research sequences selected for their potential to improve the characterization of mixed pathologies. This initiative will initially focus on T1‐weighted structural MRI and arterial spin labeled (ASL) perfusion MRI, but other approaches can also be considered. Although there is an added logistical burden to the incorporation of research MRI sequences, nearly all sites expressed an interest in participating in this aspect of CLARiTI when surveyed.

Morphometric measures derived from T1‐weighted structural MRI provide the most commonly used imaging biomarkers of neurodegeneration. Given the known deleterious impact of motion on neurodegeneration quantification^24^, ^25^, ^26^, ^27^ and selection bias from disease‐related motion quality control (QC) segregation,^25^ motion‐corrected anatomical imaging may improve the precision for quantifying regional volumes and cortical thickness in key brain regions of interest for detecting longitudinal changes in MRI‐defined neurodegeneration. Motion corrected (MoCo) magnetization‐prepared rapid gradient echo (MPRAGE) using either prospective^27^ or retrospective^24^ approaches are now a potential alternative to the standard ADNI/SCAN MPRAGE sequence. In CLARiTI, prospective volumetric navigator (vNAV) motion‐corrected MPRAGE will optionally be collected on Siemens and Phillips scanners, while on GE scanners, MPnRAGE will optionally be collected for retrospective motion‐correction. By comparing findings from motion‐corrected scans to the standard SCAN T1‐weighted MPRAGE, these additional sequences will allow both the extent of participant motion, including group effects, and the degree to which MoCo data improve quantitative estimates of gray matter metrics to be assessed.

ASL perfusion MRI provides MRI‐based quantification of regional cerebral blood flow (CBF). Brain perfusion is of interest in mixed pathologies because CBF reflects not only cerebrovascular integrity but also regional neural activity and neurodegeneration owing to the tight coupling of CBF with regional neural metabolism.^28^ Evidence accumulated over the past two decades demonstrates that ASL CBF detects regional hypoperfusion in ADRD, can predict clinical or cognitive decline as well as disease conversion, and may be one of the earliest biomarkers to change in the AD continuum.^29^, ^30^, ^31^, ^32^, ^33^, ^34^, ^35^, ^36^ Studies comparing ASL to fluorodeoxyglucose (FDG) in ADRD also show analogous patterns of change and similar sensitivity and specificity for neurodegeneration.^37^, ^38^, ^39^, ^40^, ^41^, ^42^ As vascular contributions to dementia are common,^43^ we note that reductions in CBF are also reliably detected with ASL.^44^, ^45^, ^46^, ^47^ Although dozens of studies support ASL MRI as a biomarker in ADRD, most existing data were acquired from small convenience samples and/or using now outmoded methods. CLARiTI will support the use of background‐suppressed pseudocontinuous ASL acquired with a segmented 3D readout optimized to maximize spatial resolution and minimize susceptibility artifact. Emerging velocity‐selective ASL methods^48^ will also be evaluated, and a multi‐postlabeling delay acquisition will be made available for multicompartmental modeling, which could improve the accuracy of CBF quantification. Features enabling improved and site‐harmonized ASL that are not yet widely deployed in ADRD research will be evaluated in CLARiTI.

### 2.4 FDG PET

FDG PET measures glucose metabolism and is an optional procedure within CLARiTI that can be collected on participants with MCI or dementia. Patterns of FDG may be useful for predicting subgroups that reflect different underlying etiologies as well as combinations of co‐pathology.^49^ FDG can also be leveraged to validate “synthetic” FDG PET derived from ASL and structural MRI.

### 2.5 Blood

Blood will be collected proximally to the time of PET using NCRAD‐supplied kits and processed by the site with a uniform preanalytic protocol. The entire process will follow the standard operating procedures for the AD fluid biomarker initiative (ADCFB) on the NCRAD website. Approximately 30 mL of blood will be collected in ethylenediaminetetraacetic acid vacutainers. The sample is gently inverted 8 to 10 times and centrifuged at 2000 g for 10 minutes at 4°C. The buffy coat is removed from the sample as a source of DNA and immediately frozen in dry ice or at −80°C. Plasma is aliquoted in 0.5 mL increments into polypropylene cryovials and immediately frozen at −80°C. The time from draw to freezer is ≈ 60 to 80 minutes. Meta‐data regarding the blood draw are collected on a standard flow sheet including a recording of the time and content of the last food intake (fasting is not required), time of start and stop of the blood draw, and timing of processing steps.

Samples are shipped to NCRAD in NCRAD‐supplied shipping kits for central management and coordination of assays. DNA will be extracted from the buffy coat. A DNA single nucleotide polymorphism (SNP) fingerprint that includes the two SNPs required for *APOE* genotyping, will be completed. At NCRAD, a genome‐wide SNP array may be obtained, and the remaining DNA will be made available to approved researchers. SIMOA or other immunoassay methods are planned for phosphorylated tau, amyloid beta (Aβ) peptides, alpha‐synuclein, markers of astroglia processes, and neuronal degeneration. Assays will be run in batches as they accrue (e.g., on a quarterly or semi‐annual basis). Additional exploratory assays for soluble oligomeric forms of Aβ and alpha‐synuclein may also be measured as methods are optimized. The remaining aliquots will be banked and allocated by request for mission‐aligned purposes. The collection procedure is described in detail in the NCRAD standard operating procedures for the ADCFB protocol.

We will also pilot a bloodspot procedure that will include an additional small sample of blood collected via a finger stick or similar method. Appropriately trained personnel will conduct the finger stick using a lancet and will drop two to four drops of blood onto a filter paper bloodspot cartridge. Eventual analysis of this bloodspot data will be performed with emerging technologies as they are optimized for this purpose.

### 2.6 Amyloid and tau PET visual reads

PET visual reads will be overseen by G.R., and will build on procedures already in place in LEADS and ADNI4. For CLARiTI, visual interpretations will be performed by a team of ≈ 10 trained clinicians board‐certified in neurology, radiology, nuclear medicine, or other applicable specialties, recruited from different ADRC sites. The inclusion of raters across different ADRC sites has the benefit of easily incorporating site‐level expertise, as visual reads are currently already in place across multiple ADRCs. This also provides an opportunity for site‐level training on how to perform visual reads in the context of providing research results.

For amyloid PET visual reads, raters will be required to complete vendor‐provided training as applicable (florbetaben [FBB] and florbetapir [FBP]). The same general approach detailed in these vendor‐specific training modules will be applied to the radiotracers NAV‐4694 and PiB. The method proceeds as follows: First, exams that have been uploaded from the sites are accessed at a central repository and are read by one of the certified raters as “amyloid positive” or “amyloid negative”—blind to participant clinical information and the numerical quantification result (global SUVR and CL values^22^). Next, the rater reviews the derived numerically quantified values to determine whether their visual read is concordant with quantified values. If the visual read and quantitative assessment are concordant, then the scan is designated as amyloid positive/negative as appropriate. When there is discordance between the visual read and quantitative value, scan interpretation is deferred to a consensus conference including three or more members of the clinical interpretation team. Raters can also designate any exam for consensus based on ambiguity in interpretation, even if visual reading and quantification are concordant. The central read report that is provided back to the site will include both quantification and clinical visual reads, as well as consensus information if applicable. Importantly, this report can be used as part of the disclosure process, depending on site‐level procedures for the disclosure of research results (see Disclosure section below).

Tau PET reads will follow a similar workflow that incorporates both visual reads with quantitative values. However, we acknowledge that there is less consensus in the field regarding the appropriate format of a tau PET result. In particular, regional information captured with tau PET is likely more clinically relevant than amyloid PET, suggesting a binary tau PET read may be insufficient. Further, recent clinical trial data suggest differential effects of treatment as a function of moderate versus high tau PET burden,^50^ suggesting more granular information may be needed for a clinical decision that incorporates tau PET in the future.

As part of CLARiTI, we will pilot a four‐tier approach that adheres to the criteria described in the most recent framework manuscript describing AD as a biological disease.^51^ These criteria conceptualize currently available biomarkers into two categories (Core 1 and Core 2), based on the timing of when the biomarker becomes abnormal. Core 1 biomarkers measure initial change that emerges around the time of amyloid PET positivity and includes many biofluid measures (CSF and plasma Aβ42, phosphorylated tau [p‐tau]217, p‐tau181, and p‐tau231). These Core 1 markers are sufficient for indicating whether an individual is on the AD disease spectrum. Core 2 markers change later in the disease course and provide information on disease progression (tau PET, total tau, and newer markers such as pT205). Together, Core 1 and Core 2 biomarkers describe four biological stages of AD (A, B, C, and D). Although multiple Core 2 markers are discussed in the new framework, tau PET is the only Core 2 biomarker with sufficient validation at this point and is used to define biological stages A through D.^51^

Biological stage A reflects an initial stage of pathological accumulation, defined by the presence of amyloid positivity without evidence of tau PET accumulation. Stage B includes amyloid positivity in conjunction with focal medial temporal lobe (MTL) tau PET uptake, with subsequent stages including either moderate (Stage C) or high (Stage D) neocortical tau PET signal. The standard established methods for reading 18F‐flortaucipir (FTP) will be adapted to integrate these four levels of tau burden (tau PET−, tau PET+ in the MTL only, moderate tau PET+ in the neocortex, and high tau PET+ in the neocortex). This four‐level system will also be applied to both 18F‐PI‐2620 and 18F‐MK‐6240. Similar to the amyloid interpretation method, the tau interpretation will integrate visual reads and standard image quantification, with scans flagged for consensus by the clinical raters if visual reads and quantification are incongruent, or if scan interpretation is ambiguous. Similar to amyloid PET, this resulting tau PET report can be used as part of the disclosure process, depending on site‐level procedures for the disclosure of research results (see next section).

### 2.7 PET disclosure

The return of PET and biofluid results to ADRC participants has become increasingly common, especially since the US Food and Drug Administration (FDA) approval for FBP in 2012^52^ and FBB in 2014^53^ to evaluate the presence of underlying amyloid plaque pathology in patients with clinical impairment. In a survey of 30 ADRC sites conducted in 2019 and published in 2021,^54^ 43% of sites surveyed reported disclosing amyloid PET status to impaired individuals and 27% reported disclosure of this information to cognitively unimpaired individuals. Furthermore, 10% of sites disclosed tau PET results to impaired individuals and 7% to cognitively unimpaired individuals. Amyloid and tau PET disclosure was motivated by the direct requests of participants for their data to inform a range of potential personal benefits (i.e., informing health care or medical decision‐making, advanced planning). These data mirror various studies that demonstrate participants’ strong desire for their individual research results;^55^, ^56^ in fact, many participants feel that individual result disclosure is a fundamental “right” in research.^57^ In contrast, the 2019 survey revealed that the ADRCs’ main barrier to amyloid PET disclosure was that the study consent specifically forbids disclosure (i.e., releasing individual results was not part of the original study infrastructure). For tau PET, the main barriers were that the tau PET did not meet clinical standards (not FDA approved) and that the results were not medically actionable. Additionally, the challenge of returning individual biomarker results was hindered by a lack of centralized or standardized biomarker disclosure protocols; rather, disclosure was implemented in a site‐ or study‐specific manner.^58^ These 2019 survey results provide key insights into the local behavior of ADRC sites regarding the return of PET results.

Since this 2019 survey, a number of advancements have occurred in the field that will likely increase site interest in PET result disclosure. Specifically, we have seen the first approvals of anti‐amyloid therapeutics^59^ and one tau PET ligand (FTP) has been approved by the FDA for in vivo assessment of neurofibrillary tangles.^60^ It is expected other tau PET ligands that are widely used in research (PI2620, MK6240) will also be FDA approved in the near future. Further, tau PET levels within amyloid‐positive participants have been used to stratify participants in anti‐amyloid clinical trials, with some data suggesting higher treatment effects in moderately elevated versus highly elevated tau PET subgroups.^50^ We anticipate that tau PET results will become an important consideration for medical decision making, which has important implications for disclosure efforts. Additionally, various frameworks,^61^ best practices guidelines, and detailed considerations^62^, ^63^ have been published to support an empirically validated approach to disclosure of individual biomarker results in research settings.

The Disclosure Core within CLARiTI is led by A.R.F. and L.C. and will focus on both understanding current practices across ADRCs, as well as providing resources for both amyloid and tau PET results disclosure. Given that many sites are already performing their own disclosure procedures,^54^ the Disclosure Core will work with sites to understand how CLARiTI visual read reports and disclosure resources intersect with their local practices. For instance, some sites will incorporate visual read reports into their already established disclosure protocols. However, we anticipate that many sites not currently engaged in or less familiar with disclosure will benefit from the training, protocol, and resources developed by the CLARiTI Disclosure Core.

Given the rapid development and progress of both AD biomarkers and disclosure science, the Disclosure Core will use an updated Disclosure Practices Survey to better gauge the practices and needs of the ADRCs regarding releasing PET and other biomarker results. This survey will not only evaluate parallel domains as those assessed in the 2019 Roberts survey, but also gather detailed information about differing disclosure protocols and collate tools from currently disclosing sites. These resources will be added to the Disclosure Toolkit, a collection of training materials, forms, and guidance for disclosure across the ADRCs. The Disclosure Core will use an iterative development approach to develop these resources, starting with piloting the Disclosure Toolkit in a subset of five sites selected for their experience with disclosure, geographic region, and diversity of participants served. A preliminary toolkit will then be made available to interested sites via NACC, with measures of efficacy, feasibility, and acceptability/satisfaction embedded to allow for further evaluation and updating. Disclosure Core efforts will initially focus on amyloid PET disclosure, given that amyloid PET disclosure practices are more widely accepted across ADRC sites and are also currently in place across multiple clinical trials and observational studies. Tau PET disclosure protocols will also be developed during the second half of the CLARiTI grant. Given the wide array of data being collected and disclosed at individual sites, the Disclosure Core will also explore standardized protocols for the disclosure of additional biomarker information, including plasma biomarkers.

#### 2.7.1 Disclosure protocol

CLARiTI‐specific disclosure procedures will include participant consent to learning results. Various studies have demonstrated the importance of pre‐disclosure education in preparing participants to receive their PET results and to consider risks of disclosure, which include psychological distress, experiences of stigma, and medicolegal discrimination.^64^, ^65^ Therefore, while the disclosure consent form is embedded within the CLARiTI consent form, participants will be encouraged to sign the disclosure consent form only after they have ample time to review key pre‐disclosure education materials. These materials review what information will and will not be disclosed, the meaning and limitations of this information, and the potential risks and benefits of disclosure. It is also essential to consider whether the participant is psychologically prepared to receive a biomarker results. Prior to disclosure, study staff may administer brief psychological screens to ensure no untreated clinically significant depression, anxiety, or suicide ideation is present. Participants that do not have emotional readiness will be excluded from the disclosure procedure and may be rescreened at a later time (e.g., when their symptoms have abated or stabilized, when they are actively engaged in mental health services). The results disclosure visit will be conducted with a clinician (doctor, clinical psychologist, nurse practitioner, social worker, or other qualified team member with training to whom the site principal investigator has delegated this duty) and may occur in person or remotely via Health Insurance Portability and Accountability Act–compliant televideo software or telephone. Participants will be encouraged to bring a loved one to the visit if they prefer. The clinician will assess readiness to learn results, provide the PET visual read research result, and answer questions and provide appropriate resources as needed. Brief post‐disclosure measures will assess comprehension of and reactions to results. A member of the study staff may telephone the participant to check in and assess any psychological distress or resource needs. This follow‐up phone call may be waived by the participant and/or clinician if deemed low risk or adequate support is deemed to already be in place. In addition to participant measures, sites will record additional information regarding feasibility and resource use as part of disclosure; these data will help inform the science of implementation and dissemination of biomarker disclosure not only throughout the ADRC network, but also across other research settings and in clinical practice.

### 2.8 Inclusion

Using a culturally informed, community‐engaged research approach (CI‐CER; see Rivera Mindt et al. in this issue),^66^, ^67^, ^68^ M.R.M. and O.O. will lead our CLARiTI Inclusion Core team, which will work closely with all ADRCs and community partners to support inclusive participation and engagement of historically URPs, including persons from ethnoculturally minoritized groups (e.g., Black/African American, Latinx, American Indian/Alaskan Native, Asian/Native Hawaiian/Pacific Islander), low education (≤ 12 years of education), and/or rural populations. Biomarker studies in the ADRD space have traditionally consisted of homogeneous, highly educated, non‐Latinx White individuals, greatly limiting the generalizability of current research findings related to ATN profiles. Further, studies examining biomarker profiles across URPs have yielded equivocal findings in patterns of amyloid positivity.^69^, ^70^, ^71^, ^72^ Further, it is unclear whether previously reported differences across ethnocultural groups reflect underlying biological differences or are confounded by selection procedures, geographical differences, and/or willingness of URPs to participate in biomarker studies.^73^, ^74^ It is also important to note that nearly all studies suffer from small samples of participants from URPs. Overall, it is of utmost importance to ensure our ATN studies are inclusive.

As mentioned above, inclusion into CLARiTI will focus on Clinical Core participants already enrolled in their ADRC. However, we anticipate that CLARiTI will ultimately comprise a blend of newly enrolled ADRC participants in conjunction with participants who are already actively involved in their ADRC. The Inclusion Core will focus on supporting efforts related to CI‐CER–based inclusion and engagement efforts of individual URPs in ADRD research, working with sites as needed to meet these goals. As a program, 27% of active ADRC participants are from URPs, based on self‐identified ethnocultural status. However, examination of site‐level data shows a large degree of variability in participant characteristics across sites, reflecting differences in local CI‐CER expertise and inclusion efforts (Figure 2). We anticipate that the CLARiTI Inclusion Core resources will also aid in the new enrollment of individuals from URPs into the ADRC independent of additional enrollment into CLARiTI (in other words, individuals that will enroll in an ADRC, but are not willing to complete the imaging procedures required in the CLARiTI study). A general increase in the inclusion of URPs into ADRC Clinical Cores is valuable even in the absence of enrollment into CLARiTI because it will improve the generalizability of other datatypes collected at each ADRC. Further, it is possible that individuals from URPs may subsequently become interested in ATN phenotyping at a later time point. Thus, we anticipate these efforts to have positive downstream effects, even if the direct impact on CLARiTI is not immediate.

The Inclusion Core will use an innovative, multipronged, evidence‐based approach to implement a scalable inclusion and engagement plan to assure that CLARiTI meets our participatory inclusion enrollment goals of ≥ 25% URP representation in our sample. Although this goal of ≥ 25% URP inclusion aligns with the current composition of the ADRC program (Figure 2), it is possible that the participant burden associated with three imaging visits will impact our ability to include individuals from URPs. In addition to implementing the methods described below, we will work closely with individual sites to understand barriers to participation.

Our multipronged inclusion and engagement plan includes: (1) community partnerships (e.g., grassroots studies and registries, national organizational partners, and local community‐based organizations [CBOs]), (2) a dedicated Inclusion Core team of investigators and staff, especially our community research liaisons (CRLs) and community research navigators (CRNs), (3) CER‐based marketing and digital engagement, and (4) financial incentives and transportation to/from visits for all participants. Our CI‐CER efforts will be guided by our Community‐Science Partnership Board (CSPB) and implemented by our culturally diverse and culturally competent CLARiTI Inclusion Core team.

Each CLARiTI ADRC site will be provided funding to hire approximately one full‐time CRL who will promote the inclusion of URP participants and will be responsible for active and ongoing community engagement; liaising with the Outreach, Recruitment, and Engagment (ORE) and Clinical Cores of their center as well as CBOs and community members. In addition, the CRL will provide each URP participant with wrap‐around navigational/concierge support for the in‐person study visits. Our Inclusion Core team will be responsible for their training to conduct CI‐CER and to better understand and tailor these efforts to their local communities. Training curricula for CI‐CER and cultural competence (e.g., webinars, videos, resources) will also be developed and made available to the ADRCs. In terms of workflow, CLARiTI CRLs will work closely with our community partners to inform local study inclusion/engagement efforts, earn the trust of community members, provide information and resources, and serve as a bridge between the community and ADRCs.

In addition to CRLs at the site level, there will be up to five CRNs within the Inclusion Core proper. These positions build on the Care Navigator models to provide more intensive support to maximize the engagement of participants and study partners from diverse backgrounds. Our full‐time CRNs will work virtually (e.g., phone and/or online) and be centrally located in New York City and Madison, Wisconsin, and will be co‐supervised by our Inclusion Core leadership team. They will work closely with each ADRC site, particularly with the site's CRL, to support participants’ engagement in all components of the study. A key role for our CRN team will be to maintain active cohort engagement throughout the 5‐year study period. Specifically, CRNs will promote retention and task completion during each participant's “active phase” of study participation as they transition through the different study visits, as well as to continue to foster trust, and offer interested participants referral to other trusted NIA‐funded studies and clinical trials via opt‐in options selected during consent to study. Longitudinal engagement activities for all participants will include quarterly newsletters, annual surveys, mailings and e‐mails for birthdays and study anniversaries, and ongoing social media engagement.

Last, our CLARiTI CSPB is currently being convened and will help to guide the efforts of the Inclusion Core team to deploy our CI‐CER–based efforts. A key aspect of this work will be for our CSPB and Inclusion Core team to closely collaborate with a well‐established marketing firm that has extensive experience in URP‐focused inclusion and engagement in ADRD research. Together, we will develop a comprehensive CI‐CER–based inclusion/engagement campaign that is culturally tailored (e.g., language [English and Spanish], images) and positive. This marketing and engagement campaign will include culturally tailored engagement tools, social media content, and advertising that will connect with URP adults and inform them about the benefits and considerations for engaging in this study. Non‐digital recruitment approaches will also be developed and tailored by site and will include engagement through investigator interviews in the media, health fairs, community events, and educational resources.

## 3 ANTICIPATED IMPACT

The overarching goal of CLARiTI is to enhance the national ADRC program by providing a fundamental imaging–biofluid resource focused on mixed pathologies in a more representative sample than what has been accomplished thus far in the field (such as with studies more narrowly focused on amnestic AD). This effort is the first coordinated, protocol‐driven, imaging–biofluid effort that involves all ADRC sites. By leveraging comprehensive ATN imaging and biofluid data on expertly characterized Clinical Core participants spanning the spectrum of ADRD, we aim to derive etiologic profiles reflecting common neurodegenerative diseases.

### 3.1 Etiological signatures

We will evaluate a variety of analytic approaches to characterize disease signatures in the context of mixed pathology, including classification algorithms (Table 2) such as subtype and stage inference (SuStaIn),^75^, ^76^, ^77^, ^78^ latent time latent class joint mixed effect (LTLCJMM),^79^, ^80^ mixture of experts (MoE),^81^, ^82^ StateViewer,^49^ as well as pathology‐informed template matching procedures to capture MRI and FDG PET–based signatures of neurodegeneration. Despite the lack of direct markers of non‐AD proteinopathy, there is strong evidence that A and T information combined with spatial patterns of N can be leveraged to estimate the likelihood of co‐pathology. For instance, a residual approach that captures the degree of neurodegeneration relative to co‐local tau PET burden has been used to classify individuals who may have additional co‐pathologies (greater neurodegeneration than predicted by tau) or may show elevated resilience (less neurodegeneration than predicted by tau).^18^, ^83^ Excitingly, this approach has also shown promising correspondence with underlying neuropathology. Specifically, a “limbic‐vulnerable” group defined with *ante mortem* MRI and *post mortem* measures of tau displayed increased MTL TDP‐43 pathology.^84^ A related study found that patterns of neurodegeneration are probabilistically linked to non‐AD pathologies even in the presence of AD. Those with neuropathologically verified TDP‐43 exhibited greater anterior than posterior MTL atrophy in cases with *ante mortem* MRI.^85^ Overall, this work highlights the utility of comprehensive ATN profiling not only to capture underlying AD etiology, but to make inferences on the presence of additional common co‐pathologies. One important limitation we anticipate is that although we will prioritize the inclusion of individuals from URPs, we will likely lack the statistical power to understand whether etiological signatures differ by subgroups based on self‐reported race and ethnicity.

**TABLE 2:** Summary of proposed analytic approaches.

Method	Inputs	Outputs	Use context
A,T PET standard classification	Scaled images for visual read; Centiloid > 20, Centaurs > 20 etc.	A status; T status; Centiloid levels Broad classes: A+,T+ = AD; A−T− = non‐AD or none; A+,T− = AD continuum	A/T categorization as explained by Jack et al.{Jack, 2018 #802} May be used in conjunction with methods below to define non‐AD and AD & co‐pathology
Subtype and Stage Inference (SuStaIn)	Multimodal imaging ROIs; A,T status; training set may be informed by neuropath‐derived disease specific MRI patterns	A disease type and stage ordinal score for each person for each class: Broad classes: AD&, non‐AD, none Specific level: AD, LBD, LATE, others, none	Cross‐sectional. Useful for identifying types or classes of etiology as well as within‐disease subtypes and ordinal stages of progression
Latent time latent class joint mixed effect models	A, T PET and MRI ROIs; requires longitudinal data	Probability of class membership based on pattern of progression. Because AD is known from A and T PET, the method will be trained on LBD and LATE latent classes	Longitudinal. Class is inferred from relative timing of atrophy and pathology patterns. Provides biomarker curves for subtypes
Mixture of experts (MoE)	Panel of MRI, A and T PET, FDG features, assay results	Probability of membership in clusters of: AD, non‐AD, AD& (mixture), no pathology	Cross sectional. Can flexibly use image and other data inputs (genetics, clinical, or other classifier output)
StateViewer	FDG + other modality ROI or summary metrics (e.g., Centiloid, Centaur, QSM, CTh)	Log odds score for each of several pathology categories for each person: Broad level: AD, non‐AD, AD&, none Specific level: AD, LBD, LATE, others, none	Cross‐sectional: applicable when FDG scans are available alone or in addition to any combination of A,T and MRI quantitative features

Abbreviations: A, amyloid; AD, Alzheimer's disease; FDG, fluorodeoxyglucose; LATE, limbic‐predominant age‐related TDP‐43 encephalopathy; LBD, Lewy body dementia; MRI, magnetic resonance imaging; PET, positron emission tomography; QSM, quantitative susceptibility mapping; ROI, region of interest; T, tau.

### 3.2 Data integration

CLARiTI will leverage established infrastructure to support protocol‐driven science focused on ATN imaging and characterization of mixed pathologies across ADRC sites. One defining feature of CLARiTI is the implementation of SCAN‐compliant PET and MRI protocols, allowing CLARiTI data to be easily mergeable with additional incoming imaging data that is collected throughout the ADRC program on non‐CLARiTI participants. As mentioned earlier, we anticipate 1600 to 1700 Clinical Core participants per year to be characterized with ATN imaging across efforts within the ADRC program (4000–5000 unique Clinical Core participants by the end of the CLARiTI project; Figure 3). As SCAN‐compliant protocols are identical to imaging acquisitions currently in place for ADNI, all CLARiTI, SCAN, and ADNI imaging data can be combined by investigators for increased statistical power, validation, and comparison of effects in the context of mixed pathologies (SCAN, CLARiTI) versus more traditional AD clinical presentations (ADNI). In addition to these standardized imaging acquisitions, there are opportunities to also leverage NACC imaging data collected through site‐specific protocols prior to the launch of SCAN, which is a focus on ongoing harmonization efforts such as the Alzheimer's Disease Sequencing Project–Phenotype Harmonization Consortium (ADSP‐PHC, U24 AG074855, led by T.H.). The ADSP‐PHC focuses on post hoc harmonization of genetic ADRD datasets and is currently evaluating the ability to combine imaging phenotypes across studies implementing different acquisition protocols (such as NACC, ADNI, A4, WRAP, HABS‐HD, OASIS, etc.). This will enable large‐scale discovery of novel genetic factors by leveraging quantitative phenotypes that can complement discoveries made with a traditional case–control study design. Overall, we anticipate that CLARiTI data will not only contribute to addressing ADRC‐specific research questions, but also to multiple efforts focused on leveraging data within and beyond the ADRC program.

### 3.3 Plasma validation

CLARiTI's approach provides an ideal platform for accelerating biomarker validation and discovery. The discovery of blood‐based markers for common co‐pathologies in ADRD has been slowed due to a lack of *ante mortem*–collected blood samples matched to standardized neuropathology of well‐characterized tissues or high‐quality *ante mortem* imaging techniques that can specifically and sensitively identify each individual co‐pathology. CLARiTI will provide this resource. We will assay cutting‐edge markers of ADRD and also create a resource for biomarker discovery and testing in the context of mixed etiologies. CLARiTI is well positioned to contribute to the validation of these new emerging markers against imaging profiles suggestive of co‐pathology.

The use of imaging profiles to detect etiologies beyond hallmark AD proteinopathy is especially relevant for clinical trial participant selection and study precision. When there is undetected mixed concomitant pathology accompanying AD proteinopathy, the interpretation of the effect of single‐etiology therapy targeting only the known and not the *unknown* causes of impairment may be confounded^2^, ^86^, ^87^ and confounding of clinical trial outcomes presuming single etiologies.^88^ This has hindered progress in identifying *ante mortem* constituent pathologies in individuals, and their progression patterns. Such knowledge would greatly inform clinical trial participant selection, improve diagnosis and prognosis, and accelerate biomarker and treatment discovery. Although tremendous progress has been made to understand the natural history of disease progression across multiple large‐scale ongoing cohort studies, these approaches are typically designed around *one* etiologic pathway or clinical syndrome and are likely excluding a large degree of heterogeneity that likely exists in real‐world patients with cognitive impairment.

### 3.4 Neuropathology

CLARiTI leverages ADRC pipelines for consenting participants for brain donation, and the robust neuropathology core network in the ADRC program. Neuropathology efforts in CLARiTI will be led by D.K. In preparation for CLARiTI, we examined “legacy” imaging–neuropathology datasets already available on NACC participants and confirmed the high prevalence of co‐pathology among Clinical Core participants who were clinically diagnosed with AD and underwent imaging at their site (Table 3). We expect similar levels of co‐pathology in CLARiTI participants, and can use this legacy imaging–neuropathology dataset to start building imaging templates that reflect patterns of atrophy associated with different pathologies (Table 2).

**TABLE 3:** Co‐pathology in NACC.

	Clinically suspected AD (*N*, %)	Normal cognition (*N*, %)
**Absent pathology**		
T− / LB− / TDP43−	15 (6%)	39 (83%)
**Single‐pathology**		
T+ / LB− / TDP43−	138 (58%)	3 (6.4%)
T− / LB+ / TDP43−	4 (1.7%)	1 (2.1%)
T− / LB− / TDP43+	9 (3.8%)	1 (2.1%)
**Co‐pathology**		
T+ / LB+ / TDP43−	18 (7.6%)	1 (2.1%)
T+ / LB− / TDP43+	45 (18.9%)	2 (4.2%)
T− / LB+ / TDP43+	1 (0.4%)	0
**Poly‐pathology**		
T+ / LB+ / TDP43+	8 (3.4%)	0
**Total with all three *post mortem* measurements**		
	238 (100%)	47 (100%)

*Notes*: Using the March 2022 NACC data freeze (“investigator_nacc57.csv”), we identified 943 individuals with MRI and *post mortem* data available on NACC. We sought to understand the occurrence of co‐pathology among individuals diagnosed clinically with AD that also participate in imaging studies. We focused on three common pathological processes relevant to clinical impairment: Neurofibrillary tangles, Lewy bodies, and TDP‐43; 581/943 cases had AD listed as a primary suspected etiology underlying cognitive impairment at their last clinical visit (of these, 518 had dementia, 58 with MCI, 1 with impaired‐not‐MCI status, and 4 with unknown cognitive status). Additionally, we identified 99 individuals with normal cognition closest to death that also had MRI data and *post mortem* data. Of the total sample of suspected AD and normal controls, 238 with suspected AD and 47 normal controls had all three pathological processes measured. Tangle+ (T+) was defined as the presence of Braak IV and higher (NACCBRAA > 3), Lewy Body+ (LB+) was defined as having diffuse LB in neocortex (NACCLEWY = 3), and TDP43+ was defined as having TDP‐43 in entorhinal cortex/inferior temporal (NPTDPD = 1).

Abbreviations: AD Alzheimer's disease; MCI, mild cognitive impairment; MRI, magnetic resonance imaging; NACC, National Alzheimer's Coordinating Center.

We estimate one to two CLARiTI participant deaths per site per year with a 65% autopsy rate for a total of ≈ 175 brains within 5 years. As centers implement new NIA ATN requirements, we will work to include deaths of ATN‐characterized clinical core participants to supplement the CLARiTI cohort. For every CLARiTI donor, we leverage standard NIA–Alzheimer's Association (AA) tissue sampling protocols across sites to develop a set of tissue samples from each donor that will be used for centralized staining, digital scanning, and quantitation to generate a quantitative neuropathology resource (whole slide imaging and data), hosted by NACC and linked to participant profiles/metadata and ATN neuropathology reference set. Centers will perform their standard neuropathology workup, compliant with NACC UDS including assessment of ADNC, LB disorders, LATE, and vascular brain injury, and send selected blocks/slides from core (NIA‐AA) regions, including dorsolateral prefrontal cortex (DLPFC), superior/middle temporal gyri (SMTG), inferior parietal lobule (IPL), calcarine cortex, posterior cingulate gyrus, amygdala, entorhinal cortex and hippocampus, basal ganglia/neostriatum, midbrain/pons, and cerebellum. Specifically, we ask that sites prepare eight additional unstained slides for CLARiTI, or fixed tissue/paraffin blocks, to send to the University of Washington (UWash) ADRC Precision Neuropathology (NP) Core, where the tissues will be stained for hematoxylin & eosin/Luxol Fast Blue (white matter/vasculature), Aβ, p‐tau, pTDP‐43, and alpha‐synuclein with all slides scanned and subjected to quantitative digital image analysis using HALO supplemented with artificial intelligence for segmentation and optimization. Neuropathological assessments will also be performed according to NIA‐AA and accepted guidelines to continue to understand and improve interrater and intersite variability and the scanned images provided for download for each center and hosted at NACC associated with NACC IDs and other data to be available to ADRCs and ADRD researchers. Leveraging this resource, and any additional ATN‐characterized ADRC clinical core participant brain donors, we will develop regular (quarterly) virtual meetings for participating NP Core leaders for their information, to review progress, and to develop analysis strategies and a consensus approach, with planned in person annual meetings in association with the Fall ADRC NP Core session to include updates on progress, slide/case/series review, and consideration of impact to current diagnostic guidelines and practice.

### 3.5 Conclusion

CLARiTI was designed to leverage ADRC infrastructure, site capabilities, as well as approaches that were developed over 20 years in ADNI related to standardized multi‐site image acquisition and analyses. CLARiTI will also benefit from innovative participation and engagement efforts that have been deployed in ADNI3 and 4 to improve the inclusion of participants from historically URPs in ADRD research. By extending this extensive and impactful experience in AD neuroimaging to the diverse and deeply phenotyped participants in ADRC cohorts, CLARiTI is well positioned to address current gaps in the in vivo characterization of mixed pathologies.

## CONFLICT OF INTEREST STATEMENT

All authors receive funding from the NIH. Additionally, E.C.M. receives funding from the Alzheimer's Association, Simons Foundation, and Archer Foundation; and has been a consultant for Eli Lilly, Biogen, Roche, and Alector. D.A.W. receives funding from Biogen and consulting fees or honoraria from Qynapse, Eli Lilly, Functional Neuromodulation, and GSK. V.L.V. receives funding from Piramal Imaging. H.R. receives funding from Biogen, and consulting fees from Wave Neuroscience, Ionis Pharmaceuticals, Eisai Pharmaceuticals, and Genentech. G.D.R. received funding from Alzheimer's Association, the American College of Radiology, Eli Lilly, Life Molecular Imaging, GE Healthcare, Genentech, and the Rainwater Charitable Foundation; consultant fees from Eli Lilly, GE Healthcare, Roche, Genentech, and Alector. S.R.L. receives consulting fees from Applied Cognition. W.J.J. receives funding from Alzheimer's Association and Roche/Genentech; and consultant fees from Eisai and Lilly. T.J.H. served on an advisory board for Vivid Genomics. M.C.D. receives funding from Eli Lilly and Eisai; and consulting fees from Roche. B.C.D. receives consulting fees from Acadia, Alector, Arkuda, Biogen, Denali, Eisai, Genentech, Lilly, Merck, Takeda, and Wave LifeSciences. J.L.D. receives funding from Eli Lilly and AstraZeneca; consulting fees to Eisai, Abbvie, Genotix Biotechnologies Inc., Gates Ventures, Karuna Therapeutics, AlzPath Inc., Cognito Therapeutics, Inc.; and is a founder of Monument Biosciences. S.C.J. receives consulting fees from Enigma and Alzpath. Author disclosures are available in the supporting information.

## CONSENT STATEMENT

Written informed consent will be obtained from all study participant or their legally authorized representative that enroll into the CLARiTI study.

## Supporting information



Supporting information
